# Update: Characteristics of Health Care Personnel with COVID-19 — United States, February 12–July 16, 2020

**DOI:** 10.15585/mmwr.mm6938a3

**Published:** 2020-09-25

**Authors:** Michelle M. Hughes, Matthew R. Groenewold, Sarah E. Lessem, Kerui Xu, Emily N. Ussery, Ryan E. Wiegand, Xiaoting Qin, Tuyen Do, Deepam Thomas, Stella Tsai, Alexander Davidson, Julia Latash, Seth Eckel, Jim Collins, Mojisola Ojo, Lisa McHugh, Wenhui Li, Judy Chen, Jonathan Chan, Jonathan M. Wortham, Sarah Reagan-Steiner, James T. Lee, Sujan C. Reddy, David T. Kuhar, Sherry L. Burrer, Matthew J. Stuckey

**Affiliations:** ^1^CDC COVID-19 Response Team; ^2^New Jersey Department of Health; ^3^New York City Department of Health and Mental Hygiene; ^4^Michigan Department of Health and Human Services.

As of September 21, 2020, the coronavirus disease 2019 (COVID-19) pandemic had resulted in 6,786,352 cases and 199,024 deaths in the United States.* Health care personnel (HCP) are essential workers at risk for exposure to patients or infectious materials (*1*). The impact of COVID-19 on U.S. HCP was first described using national case surveillance data in April 2020 (*2*). Since then, the number of reported HCP with COVID-19 has increased tenfold. This update describes demographic characteristics, underlying medical conditions, hospitalizations, and intensive care unit (ICU) admissions, stratified by vital status, among 100,570 HCP with COVID-19 reported to CDC during February 12–July 16, 2020. HCP occupation type and job setting are newly reported. HCP status was available for 571,708 (22%) of 2,633,585 cases reported to CDC. Most HCP with COVID-19 were female (79%), aged 16–44 years (57%), not hospitalized (92%), and lacked all 10 underlying medical conditions specified on the case report form^†^ (56%). Of HCP with COVID-19, 641 died. Compared with nonfatal COVID-19 HCP cases, a higher percentage of fatal cases occurred in males (38% versus 22%), persons aged ≥65 years (44% versus 4%), non-Hispanic Asians (Asians) (20% versus 9%), non-Hispanic Blacks (Blacks) (32% versus 25%), and persons with any of the 10 underlying medical conditions specified on the case report form (92% versus 41%). From a subset of jurisdictions reporting occupation type or job setting for HCP with COVID-19, nurses were the most frequently identified single occupation type (30%), and nursing and residential care facilities were the most common job setting (67%). Ensuring access to personal protective equipment (PPE) and training, and practices such as universal use of face masks at work, wearing masks in the community, and observing social distancing remain critical strategies to protect HCP and those they serve.

Data from laboratory-confirmed and probable COVID-19 cases, voluntarily reported to CDC from state, local, and territorial health departments during February 12–July 16, 2020, were analyzed. COVID-19 cases are reported using a standardized case report form, which collects information on demographic characteristics, whether the case occurred in a U.S. health care worker (HCP status), symptom onset date, underlying medical conditions, hospitalization, ICU admission, and death. HCP occupation type and job setting were added to the case report form in May, enabling prospective and retrospective entry of these elements. Case surveillance data were enriched with additional cases from a COVID-19 mortality-focused supplementary surveillance effort in three jurisdictions^§^ (*3*). Descriptive analyses were used to examine characteristics by vital status. HCP occupation type and job setting were reported by a subset of jurisdictions with at least five HCP cases for each variable. Analyses were conducted using Stata (version 15.1; StataCorp) and SAS (version 9.4; SAS Institute).

Among 2,633,585 U.S. COVID-19 cases reported individually to CDC during February 12–July 16, HCP status was available for 571,708 (22%) persons, among whom 100,481 (18%) were identified as HCP. Data completeness for HCP status varied by jurisdiction; among jurisdictions that included HCP status on ≥70% of cases and reported at least one HCP case (11), HCP accounted for 14% (14,938 of 109,293) of cases with HCP status available and 11% (14,938 of 132,340) of all reported cases. Case report form data were enriched with 89 additional HCP cases using supplementary mortality data; thus, the final HCP case total for analysis was 100,570 (Table 1).

**TABLE 1:** Demographics, underlying medical conditions, hospitalization status, and intensive care unit (ICU) status among health care personnel (HCP) with COVID-19, by vital status — United States, February 12–July 16, 2020

Characteristic*	No. (%)				Case fatality ratio,^§^ no./total no.
	Total	Alive	Deceased^†^	Unknown	
**Total**	**100,570**	**67,105**	**641**	**32,824**	**0.95 (641/67,746)**
**Age group (yrs)**	**N = 100,432**	**N = 67,023**	**N = 641**	**N = 32,768**	**—**
16–44	57,742 (57)	39,018 (58)	57 (9)	18,667 (57)	0.15 (57/39,075)
45–54	20,981 (21)	13,836 (21)	99 (15)	7,046 (22)	0.71 (99/13,935)
55–64	17,052 (17)	11,264 (17)	205 (32)	5,583 (17)	1.79 (205/11,469)
≥65	4,657 (5)	2,905 (4)	280 (44)	1,472 (4)	8.79 (280/3,185)
**Sex**	**N = 99,741**	**N = 66,796**	**N = 639**	**N = 32,306**	**—**
Female	78,328 (79)	52,366 (78)	395 (62)	25,567 (79)	0.75 (395/52,761)
Male	21,413 (21)	14,430 (22)	244 (38)	6,739 (21)	1.66 (244/14,674)
**Race/Ethnicity**	**N = 69,678**	**N = 45,104**	**N = 552**	**N = 24,022**	**—**
American Indian/Alaska Native, non-Hispanic	253 (0)	186 (0)	0 (0)	67 (0)	—
Asian, non-Hispanic	6,010 (9)	4,083 (9)	111 (20)	1,816 (8)	2.65 (111/4,194)
Black, non-Hispanic	18,117 (26)	11,172 (25)	177 (32)	6,768 (28)	1.56 (177/11,349)
Hispanic/Latino^¶^	8,030 (12)	4,262 (9)	49 (9)	3,719 (15)	1.14 (49/4,311)
Multiple/Other, non-Hispanic	4,195 (6)	2,662 (6)	13 (2)	1,520 (6)	0.49 (13/2,675)
Native Hawaiian/Other Pacific Islander, non-Hispanic	422 (1)	314 (1)	4 (1)	104 (0)	1.26 (4/318)
White, non-Hispanic	32,651 (47)	22,425 (50)	198 (36)	10,028 (42)	0.88 (198/22,623)
Underlying medical conditions**	**N = 40,582**	**N = 26,868**	**N = 378**	**N = 13,336**	**—**
Any underlying medical condition	17,838 (44)	11,012 (41)	348 (92)	6,478 (49)	3.06 (348/11,360)
Any chronic lung disease	6,422 (16)	4,064 (15)	89 (24)	2,269 (17)	2.14 (89/4,153)
Any cardiovascular disease	7,348 (18)	4,331 (16)	229 (61)	2,788 (21)	5.02 (229/4,560)
Diabetes mellitus	5,466 (13)	3,314 (12)	198 (52)	1,954 (15)	5.64 (198/3,512)
Immunosuppressing condition	1,504 (4)	1,070 (4)	24 (6)	410 (3)	2.19 (24/1,094)
Severe obesity	1,101 (3)	453 (2)	27 (7)	621 (5)	5.63 (27/480)
Chronic renal disease	503 (1)	279 (1)	45 (12)	179 (1)	13.89 (45/324)
Neurologic/Neurodevelopmental disability	528 (1)	333 (1)	34 (9)	161 (1)	9.26 (34/367)
Chronic liver disease	242 (1)	148 (1)	10 (3)	84 (1)	6.33 (10/158)
Autoimmune condition	479 (1)	262 (1)	3 (1)	214 (2)	1.13 (3/265)
Psychologic/psychiatric condition	353 (1)	191 (1)	4 (1)	158 (1)	2.05 (4/195)
**Admission to hospital**	**N = 83,202**	**N = 55,415**	**N = 591**	**N = 27,196**	**—**
Yes	6,832 (8)	4,207 (8)	518 (88)	2,107 (8)	10.96 (518/4,725)
**Admission to ICU**	**N = 33,694**	**N = 22,545**	**N = 377**	**N = 10,772**	**—**
Yes	1,684 (5)	662 (3)	295 (78)	727 (7)	30.83 (295/957)

**Abbreviation:** COVID-19 = coronavirus disease 2019.

* Variable completeness varied by case characteristic: age (>99%), sex (99%), race and ethnicity (69%), hospitalization status (83%), ICU admission status (34%); characteristic-specific sample size for cases with available information are presented for each grouping. N = number with available information.

^†^ Death outcomes were known for 67,746 (67%) HCP cases; of these, 91 additional new fatal cases were included based on data from the supplementary mortality project (89 newly identified as HCP and two newly identified deaths among known HCP). Additional available data for these 91 cases were incorporated if missing in the national case surveillance data.

^§^ Deaths per 100 HCP cases with known death status.

^¶^ Cases reported as Hispanic were categorized as “Hispanic or Latino persons of any race” regardless of availability of race data.

** Underlying medical condition status was classified as “known” if any of these 10 conditions, specified on the standard case report form, were reported as present or absent: diabetes mellitus, cardiovascular disease (includes hypertension), severe obesity (body mass index ≥40 kg/m^2^), chronic renal disease, chronic liver disease, chronic lung disease, immunosuppressing condition, autoimmune condition, neurologic condition (including neurodevelopmental, intellectual, physical, visual, or health impairment), or psychologic/psychiatric condition. Status for these conditions was “known” for 40,582 persons. Responses include data from standardized fields supplemented with data from the free text field for “other chronic disease/underlying condition” for the 10 specific medical conditions, if not originally specified.

Among HCP with COVID-19 overall, the median age was 41 years (interquartile range = 30–53 years); 79% of cases were in females. Among 69,678 (69%) HCP cases with data on race and ethnicity, 47% were in non-Hispanic Whites (Whites), 26% were in Blacks, 12% were in Hispanics or Latinos of any race (Hispanics), and 9% were in Asians. Of persons with known hospitalization or ICU admission status, 8% (6,832 of 83,202) were hospitalized and 5% (1,684 of 33,694) were treated in an ICU. Vital status was known for 67% (67,746) of HCP with COVID-19; among those, 641 (1%) died. Deaths among HCP with COVID-19 were reported in 22 jurisdictions. Compared with those who survived, decedents tended to be older (median age = 62 versus 40 years), male (38% versus 22%), Asian (20% versus 9%), or Black (32% versus 25%).

Among HCP cases with data on one or more of 10 underlying medical conditions specified on the case report form, 17,838 (44%) persons had at least one condition. The most common were cardiovascular disease (18%), chronic lung disease (16%), and diabetes mellitus (13%). The vast majority (92%) of fatal HCP cases were among HCP with an underlying medical condition. More than one half had cardiovascular disease (61%) or diabetes mellitus (52%), conditions known to increase the risk for severe COVID-19*^¶^*; 32% were reported to have both conditions (Table 1).

Six jurisdictions reported the occupation type** or job setting^††^ for at least five HCP with COVID-19 (Table 2). Among HCP with COVID-19 in these jurisdictions, occupation type was available for 59% (5,913 of 9,984) and job setting for 41% (6,955 of 17,052). Health care support workers accounted for the largest overall group of occupation types (32%), and nurses constituted the largest single occupation type (30%) (Table 2). Within this subset of HCP cases, two thirds (67%) were in persons reported to work in nursing and residential care facilities.

**TABLE 2:** Occupation type and job setting of health care personnel (HCP) with COVID-19 — six jurisdictions,* February 12–July 16, 2020

Characteristic (no. with available information)^†^	No. (%)
Occupation type (5,913)^§^	
Health care support worker^¶^	1,895 (32.1)
Nurse**	1,742 (29.5)
Administrative staff member	581 (9.8)
Environmental services worker	330 (5.6)
Physician	190 (3.2)
Medical technician	135 (2.3)
Behavioral health worker	128 (2.2)
First responder	113 (1.9)
Dietary services worker	113 (1.9)
Dental worker	98 (1.7)
Laboratorian	68 (1.2)
Occupational, physical, or speech therapist	65 (1.1)
Pharmacy worker	62 (1.1)
Respiratory therapist	44 (0.7)
Phlebotomist	25 (0.4)
Physician assistant	13 (0.2)
Other	311 (5.3)
**Job setting (6,955)^§^**	
Nursing and residential care facility^††,§§^	4,649 (66.8)
Hospital	1,231 (17.7)
Ambulatory health care service^¶¶^	804 (11.6)
Other	271 (3.9)

**Abbreviation:** COVID-19 = coronavirus disease 2019.

* Alaska, Kansas, Michigan, Minnesota, North Carolina, and Utah.

^†^ Occupation type data are included for five jurisdictions (Alaska, Kansas, Minnesota, North Carolina, and Utah) that reported occupation type for at least five HCP COVID-19 cases; occupation type data were known for 59% (5,913 of 9,984) of HCP cases in those jurisdictions. Job setting data are included for five jurisdictions (Alaska, Kansas, Michigan, Minnesota, and Utah) that reported job setting for at least five HCP COVID-19 cases; job setting data were known for 41% (6,955 of 17,052) of HCP cases in those jurisdictions.

^§^ Occupation type and job setting categories were determined either by inclusion on the CDC case report form or by manual review and categorization of free-text entries within “other, specify” fields. Free-text data were used to supplement existing categories for occupation (nurse, environmental services worker, physician, respiratory therapist) and setting (long-term care facility [including nursing home/assisted living facility], hospital, rehabilitation facility) and create new categories.

^¶^ Includes nursing assistant (1,444), medical assistant (123), and other care provider or aide (328); free-text fields were used to create new categories.

**Includes data from standardized fields (1,724) supplemented with data from free-text fields (18); types of nurses or nursing specialties are not specified.

^††^ Includes long-term care facility (including nursing home/assisted living facility) (4,424), rehabilitation facility (131), and group home (94).

^§§^ Michigan provides job setting data only for cases identified from long-term care facilities (2,800).

^¶¶^ Includes outpatient care center (422), home health care service (317), and dental facility (65); free-text fields were used to create new categories.

## Discussion

State, local, and territorial health departments voluntarily submit COVID-19 case notification data to CDC, and these critical data help provide a national picture of cases. The first report on HCP with COVID-19 using national case surveillance data in April 2020 (*2*) described characteristics of 9,282 HCP cases and 27 deaths among approximately 315,000 total cases. As of July 16, 2020, among approximately 2.5 million reported U.S. COVID-19 cases, 100,570 cases in HCP and 641 deaths among HCP with COVID-19 have been reported to CDC. Continued national surveillance is vital to evaluate the effect of the pandemic on HCP, and this update emphasizes the ongoing impact on this essential working population.

Among reported HCP with COVID-19, age and sex distributions remain comparable to those of the overall U.S. HCP workforce^§§^; however, compared with nonfatal COVID-19 cases in HCP, fatal HCP cases were more common among older persons and males. Similar to findings described in the overall population (*4*,*5*), HCP with underlying medical conditions who developed COVID-19 were at increased risk for death. Almost all reported HCP with COVID-19 who died had at least one of 10 underlying conditions listed on the case report form, compared with fewer than one half of those who survived. Asian and Black HCP were also more prevalent among fatal cases; disproportionate mortality of persons from some racial and ethnic groups among cases has also been described in the general population (*3*). Long-standing inequities in social determinants of health can result in some groups being at increased risk for illness and death from COVID-19, and these factors must also be recognized and addressed when protecting essential workers in the workplace, at home, and in the community. Ensuring adequate allocation of PPE to all HCP in the workplace is one important approach to mitigating systemic inequalities in COVID-19 risk (*6*). As the COVID-19 pandemic continues in the United States, HCP are faced with increasing fatigue, demands, and stressors. HCP who are at higher risk for severe illness and death from COVID-19 should maintain ongoing communication with their personal health care providers and occupational health services to manage their risks at work and in the community.

In this update, most HCP with COVID-19 were reported to work in nursing and residential care facilities. Large COVID-19 outbreaks in long-term care facilities suggest that transmission occurs among residents and staff members (*7*,*8*). During the COVID-19 pandemic, multiple challenges in long-term care settings have been identified, including inadequate staffing and PPE, and insufficient training in infection prevention and control. As the pandemic continues, it is essential to meet the health and safety needs of HCP serving populations requiring long-term care. Importantly, HCP cases were also identified from a variety of other health care settings. Therefore, increased access to resources, appropriate training, and ongoing support are needed across the health care spectrum to protect all HCP and their patients.

HCP with COVID-19 were reported among a diverse range of occupations. Nurses represented 30% of HCP cases with known occupation type, but account for only approximately 15% of the total U.S. health care and social assistance workforce.^¶¶^ Nurses and health care support workers often have frequent, close contact with patients and work in settings that might increase their risk for acquiring SARS-CoV-2, the virus that causes COVID-19. HCP who do not provide direct patient care, such as administrative staff members and environmental service workers, were also reported to have COVID-19. Risk to HCP can occur through pathways other than direct patient care, such as exposure to coworkers, household members, or persons in the community. HCP who acquire SARS-CoV-2 can similarly introduce the virus to patients, coworkers, or persons outside the workplace. Thus, practices such as universal use of face masks at work, wearing masks in the community, observing social distancing, and practicing good hand hygiene remain critical strategies to protect HCP and the populations they serve. Screening HCP for illness before workplace entry and providing nonpunitive sick leave options remain critical practices.

The findings in this report are subject to at least five limitations. First, although reporting completeness increased from 16% in April to 22% in July (*2*), HCP status remains missing for most cases reported to CDC. HCP might be prioritized for testing, but the actual number of cases in this population is most certainly underreported and underdetected, especially in asymptomatic persons (*9*,*10*). Second, the amount of missing data varied across demographic groups, underlying medical conditions, and health outcomes; persons with known HCP status and other information might differ systematically from those for whom this information is not available. Third, details of HCP occupation type and job setting were not included on the CDC case report form until May 2020, and only six jurisdictions reported these data. Fourth, testing strategies and availability can vary by jurisdiction and health care setting, influencing the numbers and types of HCP cases detected. Finally, this report does not include information on whether exposure to SARS-CoV-2 among HCP cases occurred in the workplace or in other settings, such as the household or community.

As of July 16, 2020, 100,570 COVID-19 cases in HCP and 641 deaths among HCP with COVID-19 were reported in the United States. Information on COVID-19 among essential workers, including HCP, can inform strategies needed to protect these populations and those they serve, including decisions related to COVID-19 vaccination, when available. Factors such as demographics, including race and ethnicity, underlying health conditions, occupation type, and job setting can contribute to the risk of HCP acquiring COVID-19 and experiencing severe outcomes, including death. Given the evidence of ongoing COVID-19 infections among HCP and the critical role these persons play in caring for others, continued protection of this population at work, at home, and in the community remains a national priority.***

**Summary**

**What is already known about this topic?**

Health care personnel (HCP) are essential workers at risk for COVID-19.

**What is added by this report?**

HCP with COVID-19 who died tended to be older, male, Asian, Black, and have an underlying medical condition when compared with HCP who did not die. Nursing and residential care facilities were the most commonly reported job setting and nursing the most common single occupation type of HCP with COVID-19 in six jurisdictions.

**What are the implications for public health practice?**

Continued surveillance is vital to understand the impact of COVID-19 on essential workers. Ensuring access to personal protective equipment and training, and practices such as universal use of face masks at work, wearing masks in the community, and observing social distancing remain critical strategies to protect HCP and those they serve.

## Footnotes

* https://www.cdc.gov/coronavirus/2019-ncov/cases-updates/cases-in-us.html; https://www.cdc.gov/coronavirus/2019-ncov/covid-data/faq-surveillance.html; https://www.cdc.gov/coronavirus/2019-ncov/php/reporting-pui.html.

† Underlying medical condition status was classified as “known” if any of these 10 conditions, specified on the standard case report form, were reported as present or absent: diabetes mellitus; cardiovascular disease (includes hypertension); severe obesity (body mass index ≥40 kg/m^2^); chronic renal disease; chronic liver disease; chronic lung disease; immunosuppressive condition; autoimmune condition; neurologic condition (including neurodevelopmental, intellectual, physical, visual, or health impairment); and psychologic/psychiatric condition.

§ The supplementary mortality surveillance effort, which included persons with laboratory-confirmed COVID-19 who died during February 12–April 24, 2020, identified 89 additional HCP and two additional deaths among known HCP from three jurisdictions: Michigan, New Jersey, and New York City.

¶
https://www.cdc.gov/coronavirus/2019-ncov/need-extra-precautions/people-with-medical-conditions.html .

** Seventeen HCP occupation type categories: health care support worker (includes nursing assistant, medical assistant, and other care provider or aide); nurse; administrative staff member; environmental services worker; physician; medical technician; behavioral health worker; first responder; dietary services worker; dental worker; laboratorian; occupational, physical, or speech therapist; pharmacy worker; respiratory therapist; phlebotomist; physician assistant; and other; data were reported in five jurisdictions (Alaska, Kansas, Minnesota, North Carolina, and Utah).

†† Three HCP job setting categories: nursing and residential care facility (includes long-term care facility [nursing home/assisted living facility], rehabilitation facility, and group home); hospital; ambulatory health care service (includes outpatient care center, home health care service, and dental facility); data were reported in five jurisdictions (Alaska, Kansas, Michigan, Minnesota, and Utah).

§§
https://www.bls.gov/cps/tables.htm#charemp .

¶¶
https://data.bls.gov/projections/nationalMatrix?queryParams=620000&ioType=i .

***
https://www.cdc.gov/coronavirus/2019-nCoV/hcp/index.html .
